# Microbleed Detection Using Automated Segmentation (MIDAS): A New Method Applicable to Standard Clinical MR Images

**DOI:** 10.1371/journal.pone.0017547

**Published:** 2011-03-23

**Authors:** Mohamed L. Seghier, Magdalena A. Kolanko, Alexander P. Leff, Hans R. Jäger, Simone M. Gregoire, David J. Werring

**Affiliations:** 1 Wellcome Trust Centre for Neuroimaging, University College London Institute of Neurology, London, United Kingdom; 2 Stroke Research Group, Department of Brain Repair and Rehabilitation, University College London Institute of Neurology, London, United Kingdom; 3 University College London Institute of Cognitive Neuroscience, London, United Kingdom; University of California San Francisco, United States of America

## Abstract

**Background:**

Cerebral microbleeds, visible on gradient-recalled echo (GRE) T2* MRI, have generated increasing interest as an imaging marker of small vessel diseases, with relevance for intracerebral bleeding risk or brain dysfunction.

**Methodology/Principal Findings:**

Manual rating methods have limited reliability and are time-consuming. We developed a new method for microbleed detection using automated segmentation (MIDAS) and compared it with a validated visual rating system. In thirty consecutive stroke service patients, standard GRE T2* images were acquired and manually rated for microbleeds by a trained observer. After spatially normalizing each patient's GRE T2* images into a standard stereotaxic space, the automated microbleed detection algorithm (MIDAS) identified cerebral microbleeds by explicitly incorporating an “extra” tissue class for abnormal voxels within a unified segmentation-normalization model. The agreement between manual and automated methods was assessed using the intraclass correlation coefficient (ICC) and Kappa statistic. We found that MIDAS had generally moderate to good agreement with the manual reference method for the presence of lobar microbleeds (Kappa = 0.43, improved to 0.65 after manual exclusion of obvious artefacts). Agreement for the number of microbleeds was very good for lobar regions: (ICC = 0.71, improved to ICC = 0.87). MIDAS successfully detected all patients with multiple (≥2) lobar microbleeds.

**Conclusions/Significance:**

MIDAS can identify microbleeds on standard MR datasets, and with an additional rapid editing step shows good agreement with a validated visual rating system. MIDAS may be useful in screening for multiple lobar microbleeds.

## Introduction

Over the last decade, the use of iron-sensitive MRI sequences (including Gradient Echo [GRE] T2*-weighted imaging and susceptibility-weighted imaging) has increased in many clinical settings, including acute stroke units and clinics. This has led to the improved detection of cerebral microbleeds (CMBs) and other forms of intracranial haemorrhage. Cerebral microbleeds are small, rounded areas of homogeneous low signal visualized on GRE T2*-weighted images because haemosiderin (a paramagnetic product of blood degradation) has high magnetic susceptibility, causing local field inhomogeneities and signal loss. Cerebral microbleeds are due to perivascular bleeding from small vessels affected mainly by hypertensive vasculopathy and cerebral amyloid angiopathy [1]. Cerebral microbleeds are increasingly found in elderly subjects and patients with cerebrovascular disease, raising many important questions: Are patients with CMBs at increased risk of intracerebral haemorrhage? Do CMBs cause brain dysfunction? Are they a useful diagnostic marker for cerebral amyloid angiopathy (CAA)? CMBs are also found frequently in traumatic brain injury and may be a useful marker for diffuse axonal injury with potential relevance for prognosis [2]. To tackle these questions, it is important to reliably detect and map CMBs.

The current reference standard method for microbleed identification is based on the manual definition of abnormal brain tissue using visual rating scales, which is laborious, operator-dependent and time-consuming, with limited intra-rater and inter-rater reliability [3]. Furthermore, manual rating does not easily allow the comparison of the spatial distribution of microbleeds between individuals or groups, which may be crucial in assessing correlations with clinical measures, e.g. cognitive tests [4]. Because of the many CMB “mimics” with similar signal and morphological characteristics, and the unpredictable, widespread distribution of CMBs in the brain, automatic identification is challenging. Moreover, standard clinical GRE MRI inherently has limited tissue contrast and is very sensitive to susceptibility artefacts from field inhomogeneities; the modest standard field strength (1.5T), thick slices and non-isotropic voxels, may further reduce the conspicuity and size of some microbleeds [3].

An ideal automated CMB rating method should: (1) reliably detect CMBs; (2) be able to do so given standard clinical images; (3) be simple to use, and less time consuming than manual methods; and (4) should map CMBs in a common stereotactic space. We propose a new procedure for microbleed detection using automated segmentation (MIDAS), which explicitly incorporates an ‘extra’ tissue class for abnormal voxels within a unified segmentation-normalization model [5], [6]. The performance of MIDAS is assessed here on a real dataset of standard clinical MR images from 30 stroke patients.

## Results

Here, we turn to the performance of MIDAS in detecting CMBs across a range of 30 unselected patients with known CMB status based on manual identification by an expert, considered here as the reference standard to define the true positives. In the manual identification, the presence, number and anatomical distribution of CMBs was rated using the validated Microbleed Anatomical Rating Scale (MARS) [3]. The data used here were previously acquired with a standard clinical protocol and thus were not prospectively optimised for MIDAS. For more details, see the Materials and Methods section below.

**Figure 1:**
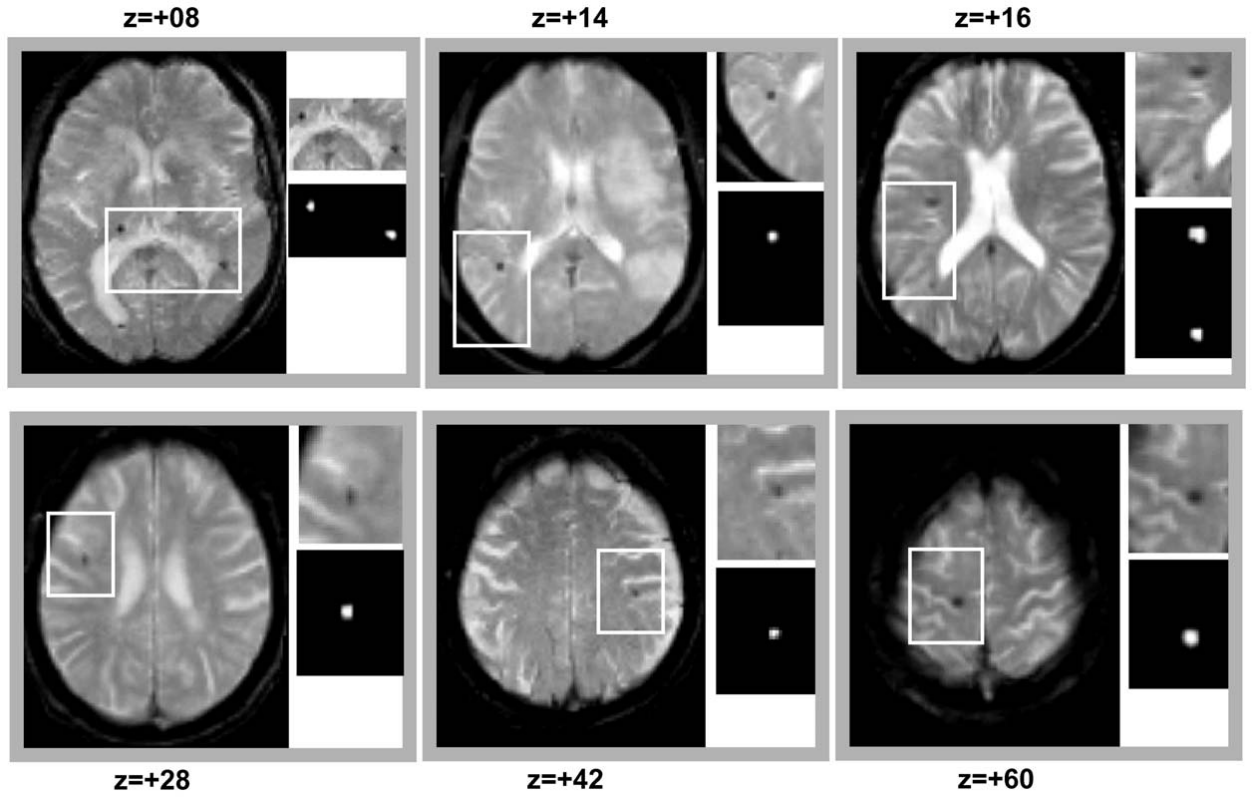
MIDAS detection in 6 different patients with variable numbers and locations of cerebral microbleeds. Typical T2* axial slices are shown with a zoom (white rectangle) on the region with successfully identified cerebral microbleed(s).

Figure 1 illustrates CMBs identified in six representative patients using MIDAS. Our method clearly identified these CMBs, of variable sizes and located in different brain regions (Figure 1).

The results can be summarised by the following key points. First, MIDAS was much more successful for detecting CMBs in lobar regions than infratentorial or deep regions (detailed results in each patient are provided in the tables in Supplementary Material S1). Specifically, across the 22 patients who have lobar CMBs, 17 patients were identified by the automated method. A close look at the 5 unsuccessful cases revealed that all of them have only a single CMB (cases 2, 8, 14, 17 and 28; see Supplementary Material S1). Thus, if we consider whether a patient has a single or multiple CMBs, we found that MIDAS identified 8 out of 13 patients (62%) who have a single CMB but successfully identified all 9 patients who have 2 or more CMBs (100%).

**Figure 2:**
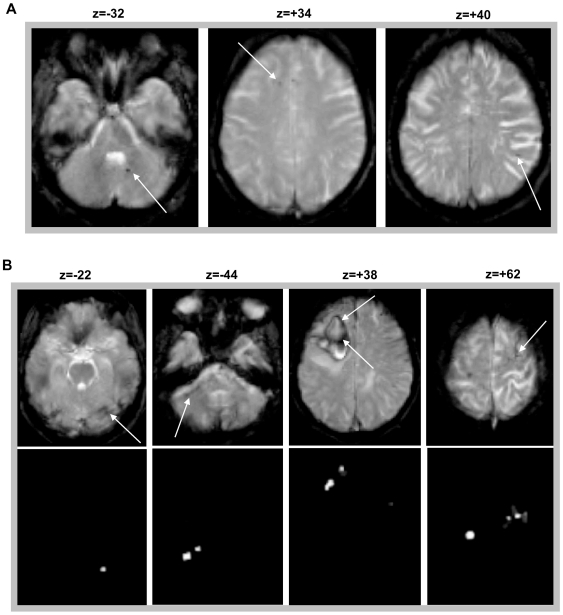
Missed cerebral microbleeds and artefacts. (**A**) example of missed cerebral microbleeds when using MIDAS (false negatives). (**B**) example of some remaining artefacts that resisted the clean-up step in MIDAS (false positives).These false positives are easily and quickly identified in a final manual editing step.

Second, some CMBs were missed by MIDAS, predominantly in two situations (Figure 2A): (i) lesions located within our artefact mask, predefined within MIDAS as a map of potential artefacts (see Materials and Methods section below), and thus rejected from the final output images (see example of a CMB in the right cerebellum, column 1 Figure 2A); and (ii) small CMBs with low contrast from their background, probably caused by partial volume effects due to the thick slices of our clinical T2* images (see columns 2 and 3 of Figure 2A).

Despite the morphological procedures applied here in cleaning up the output images (see Materials and Methods section below), the resulting maps may still contain a few artefacts. These consist of regions of low signal intensity that mimic CMBs, of three types: (i) most frequently at the edges of brain regions (air-bone susceptibility effects) – these include the inferior temporal, orbitofrontal and posterior fossa regions (see columns 1 and 2 of Figure 2B); (ii) low signal within other abnormalities in the brain such as infarcts (column 3 of Figure 2B); and (iii) flow voids in blood vessels (column 4 of Figure 2B). However, these artefacts were easily removed using a “semi-automated” approach, i.e. by (manually) excluding the obvious artefacts from the final maps. These obvious artefacts, in particular those of type (i) at the edges of brain regions, were excluded by a trained observer (MAK) and were seen in 12 patients (see the tables in Supplementary Material S1 for more details).

The Kappa coefficient for agreement between MIDAS and the reference standard manual identification (MARS) in patients who had one or more CMBs in lobar regions was 0.43, increasing to 0.65 using the semi-automated approach. The intraclass correlation coefficient for agreement about CMB count in lobar regions using MIDAS in comparison to MARS was 0.71, increasing to 0.87 when using the semi-automated method. The Kappa coefficient of identifying patients with two or more lobar CMBs increased to 0.74 using the semi-automated approach.

## Discussion

We present here a new method of identifying CMBs (MIDAS). To the best of our knowledge, this is the first (semi-)automated method developed for detecting and mapping CMBs on standard clinical images which are routinely available in many stroke clinics. Our method has several important advantages over purely manual rating: first, it eliminates intra- and inter-rater reliability; second, it is less laborious, and thus more practical for analyzing large datasets; third, MIDAS can generate detailed lesion maps in a standard coordinate space, making group analyses and correlation with clinical, behavioural or genetic data straightforward; fourth, it can automatically quantify additional spatial characteristics, including CMB location, size, and shape; fifth, the algorithm is flexible and can potentially incorporate other type of images with different contrasts and resolution. A final advantage is that the software in which our method is implemented is freely available (see Materials and Methods below).

In comparison with a validated visual rating scale (MARS), MIDAS has shown generally moderate to good agreement for the presence of lobar CMBs (Kappa 0.43, improved to 0.65 using the semi-automated method), which compares favourably with previous manual methods showing inter-observer Kappa values in the range of 0.33 to 0.88 [7]. Agreement about the number of CMBs was very good for lobar regions. Of note, our method successfully identified all patients with multiple (>1) CMBs: this may be important because multiple CMBs on standard GRE T2* sequences may be of more significance than a single lesion; it has also been suggested that only patients with more than one CMB be included in research studies, to maximize the reliability of ratings [3], [7]. The inclusion of patients judged to have a single CMB substantially reduces the inter-rater reliability of CMB identification, suggesting that raters find it most difficult to reliably decide whether one CMB or no CMBs are present. Because reliability is critical for any useful clinical or research tool, we investigated the ability of MIDAS to identify the group of patients with 2 or more CMBs. There are also biological grounds to suggest that multiple CMBs are of greater significance than a single lesion: for example, a recent prospective study of stroke patients [8] showed that a single CMB did not substantially increase the risk of ischaemic stroke or fatal intracranial haemorrhage; whilst having 2 or more CMBs did have prognostic relevance for these outcomes [8]. Thus, MIDAS could be used in a clinical or research setting to rapidly screen patients for “multiple lobar CMBs”, a group potentially of clinical importance with regard to diagnosing cerebral amyloid angiopathy (CAA) [9]; or in assessing the risk of intracerebral haemorrhage in patients taking antithrombotic drugs [8], [10].

We now discuss the potential factors that may explain the moderate agreement between the fully automated MIDAS and the manual identification using MARS. First, it is worth noting MIDAS was tested here on relatively challenging clinical data that were acquired using standard T2* sequences at 1.5T. Specifically, with a slice thickness of 5.0 mm and a gap of 1.5 mm, partial volume effects can have a strong impact on the T2* signal distribution when using the unified spatial normalisation in MIDAS that involved data smoothing and re-slicing [5], and may thus hinder the detectability of CMBs. These partial volume effects can explain why a few (tiny) CMBs have been missed by MIDAS on these datasets (as illustrated in Figure 2A). Second, the manual method (MARS) was applied on native (unresliced) images that had better in-plane resolution and were not smoothed or re-sliced, whereas MIDAS mapped all CMBs on the final normalised (re-sliced) images. Third, MIDAS operated in a monospectral mode using GRE T2* images only, whereas in the manual method clinicians were allowed to use other available images if needed to identify CMB mimics (e.g. [1]). Although this may introduce a bias in favour of the manual method, we preferred here to compare MIDAS to the manual method as commonly practiced in the clinical setting. On the other hand, we did not incorporate these additional images within the modified unified segmentation framework (for instance in a multispectral mode) because of the spatial distortion and signal loss in the T2* images that make the co-registration (voxel-to-voxel mapping) between the different images particularly challenging.

Future iterations of our technique should substantially improve its performance. For example, use of a predefined artefact mask by removing all regions with expected artefacts, might increase the specificity of our method for CMBs in deep and infratentorial regions. Importantly, the flat prior used here for CMBs (see Materials and Methods section) can potentially be further optimised if additional knowledge about the regional prevalence of CMBs is available (e.g. [11]); for instance by increasing the prior CMB probability in vulnerable regions and decreasing it in less likely regions. Furthermore, a systematic investigation of the impact of the different thresholds used here, including thresholds on posterior probabilities and size of CMBs, would help in optimizing the thresholds for specific applications more objectively. Future applications could also extend the analysis to include other imaging modalities/contrasts to better distinguish genuine CMB from their mimics.

It is premature to conclude that MIDAS should substitute manual methods for diagnosis or screening for CMBs in clinical practice. Further improvements are needed before we can recommend its routine clinical use. The limitations of MIDAS were particularly noted for patients with a single CMB (here 5 out of 13 patients with a single CMB were missed) and when using non-optimised data acquisition protocols (the output in 12 segmented patients contained some artefacts/mimics). Nevertheless, the semi-automated method with manual editing substantially improves the performance of MIDAS even on standard clinical images. For instance, to assist clinicians and speed up the detection of CMBs, it is possible to use the output from MIDAS as a starting map and then clinicians can manually alter it by either adding any missed CMBs or deleting any mimics. Manual editing using a semi-automated method has been shown to be an attractive alternative to time-consuming fully manual methods in various fields, including for instance multiple sclerosis [12], [13].

Although we have shown moderate agreement for MIDAS with the reference standard of a validated rating scale, we anticipate that it will be even more successful when applied to research-optimised data, including for instance isotropic voxels with thin slices and high in-plane resolution [14]), optimized contrast for CMB detection (e.g. susceptibility-weighted imaging [SWI] [15]), or higher magnetic field strength [14], [16].

In conclusion, this study has demonstrated a new semi-automated CMB detection method (MIDAS), which can be useful for CMB rating on standard clinical MR datasets. This method has particular promise in: (i) fast screening of patients for multiple lobar CMBs, and (ii) locating CMBs in stereotaxic space, which is useful for producing group lesion overlap maps. We plan to use this method to investigate spatially sensitive hypotheses concerning the relationship between CMBs and clinical measures, including cognitive impairment. However, further improvements in the method presented here are necessary in order to increase its performance for clinical applications.

## Materials and Methods

### Subjects

We considered unselected, consecutive patients admitted to the Stroke Service at the National Hospital for Neurology and Neurosurgery (NHNN). Our stroke service takes all suspected stroke patients admitted from the surrounding district and has a policy of performing MRI with GRE T2* sequence in all of them unless there is a contra-indication (e.g. too medically unstable, severe claustrophobia, metallic implants). Patients who did not have an MRI or with poor quality images (e.g. due to motion artefact) were excluded (30% of all patients presenting to our clinical service). The first 30 consecutive eligible patients (mean age 69 years, range 21–83 years, 14 males, 16 females) with definite or probable microbleeds on GRE T2* sequences were included (images were rated manually by an experienced observer [SMG] using the Microbleed Anatomical Rating Scale [3]). Using MARS, microbleeds were detected in 28% of our population.

We also included a control group of 44 subjects (mean age 52 years, range 21–83 years, 23 males, 21 females) referred to the Stroke Service over the same time period, who had an entirely normal MRI study (reported by a consultant neuroradiologist and checked by an experienced observer [SMG] as having no CMBs or any other imaging evidence of cerebrovascular disease). The data from these controls is not essential for the automated algorithm, but was used here to generate a map of potential artefacts (false positives) to be excluded from the output images (see below).

The study was approved by the National Hospital for Neurology and Neurosurgery Research Ethics Committee.

### Data acquisition

MRI acquisitions were carried out at 1.5 Tesla on a GE EchoSpeed system (General Electric, Milwaukee, WI, USA) using a standardized protocol, between 2004 and 2007. Each subject had axial T2-weighted Fast Spin Echo (TR = 6000 ms, TE = 105 ms, matrix = 256×224, field-of-view = 24×18 cm, slice thickness = 5 mm, slice gap = 1.5 mm, voxel size = 0.938×0.938×6.5 mm^3^, NEX2) and axial T2*-weighted gradient echo GRE (TR = 300 ms, TE = 40 ms, flip angle = 20°, field-of-view = 24×18 cm, slice thickness = 5 mm, slice gap = 1.5 mm, voxel size = 0.938×0.938×6.5 mm^3^, acquisition time 2 min).

### Microbleed Detection by Automated Segmentation (MIDAS)

All analyses were carried out with scripts written in Matlab (The MathWorks, Natick, MA, USA) that incorporated processing functions of the statistical parametric mapping (SPM8) software package (Wellcome Trust Centre for Neuroimaging, London, UK, http://www.fil.ion.ucl.ac.uk/spm/).

**Figure 3:**
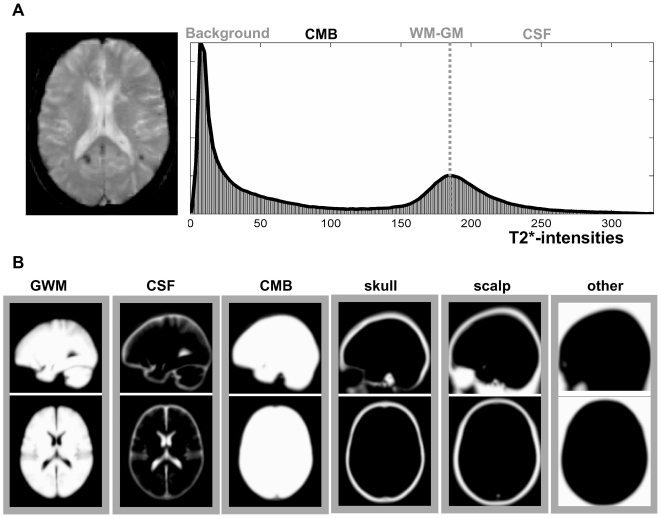
Illustrations of T2* signal intensity histogram and tissue priors. (**A**) histogram of typical T2* intensities in a patient with multiple cerebral microbleeds. The unit of T2* intensity is arbitrary. The dashed line indicates the mean intensity of gray and white tissue voxels. (**B**) Illustration of the 6 tissue priors used in MIDAS during the first iteration of unified normalisation-segmentation. GWM = gray and white matter, CSF = cerebrospinal fluid, CMB = cerebral microbleeds.

**Figure 4:**
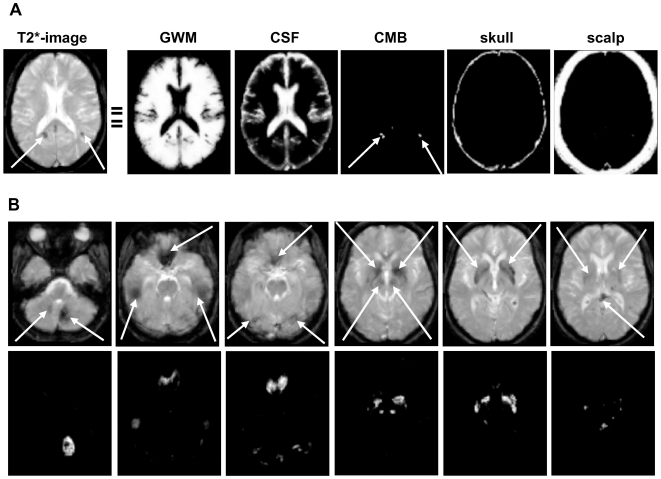
Illustrations of typical first iteration outputs. (**A**) output of the first iteration on typical T2*-images of a patient with multiple microbleeds (white arrows). (**B**) illustration of typical artefacts (white arrows) that are later removed in MIDAS.

The first step is to spatially transform or “normalize” each patient's brain images into a standard space; we used that defined by the Montreal Neurological Institute (MNI) [17]. This spatial transformation is based on the unified probabilistic normalisation-segmentation framework that combines image registration, tissue classification, and bias correction as implemented in SPM8 [5], with two major modifications: (i) it incorporates some constrains on the mixture of Gaussians used to model the T2*-weighted image intensities, and (ii) it considers CMBs as an *unexpected* and *atypical* tissue class that can explicitly be modelled within the probabilistic framework as an *extra* class with an iteratively optimised empirical prior (for more details see [6]). The output of this modified normalisation-segmentation method produces six separate probabilistic images, one for each tissue class (Figure 3B and 4A): i) normal brain, a mixture of grey and white matter (GWM); ii) cerebrospinal fluid (CSF); iii) CMBs; iv) “skull” and other low intensities around the brain; v) “scalp”; and, vi) background (other).

The initial iteration of the unified normalisation-segmentation procedure uses some modified priors and constrained mixture of Gaussians; producing a “first pass” of the empirical prior which, unlike the modified priors, is subject-specific. This subject-specific prior is optimised (i.e. cleaned up) using morphological operations on the output tissue class form the first step. This included binarisation, granulometry and masking applied on the output tissue class to reduce false positives (see pages 23 and 318 of Ref. [18]). The optimised prior is then included in a second iteration of the unified normalisation-segmentation procedure. The output is an image that codes the degree of abnormality at each voxel. Additional thresholds (on both size and height) are then used to generate a binary map that visualises CMB localisation and extent.

#### 1. Constrained mixture of Gaussians

The mixture of Gaussians used in the unified normalisation-segmentation procedure incorporates a smooth intensity variation and nonlinear registration with tissue priors [5]. The priors on the tissue class are encoded by deformable tissue probability maps generated from the averages of affine registered and tissue classified images of 452 subjects (http://www.loni.ucla.edu/ICBM/). These maps, in MNI space, represent the probabilities of finding GM, WM, and CSF tissues at each voxel. We also considered additional priors as implemented in the new segmentation toolbox of SPM8, which have been optimised for segmenting high resolution anatomical images (e.g. T1-weighted) of normal brains. The first challenge was to adapt these priors to the signal distribution in our T2*-weighted images. As illustrated in Figure 3A, the distribution of T2*-intensities has a rather poor differentiation between brain tissues, which has direct implications on the optimal choice of priors:

- GM and WM voxels appear at comparable intensities within a similar range. For this reason, the prior for coding GM and WM voxels was approximated by the sum of GM and WM priors (as one class noted GWM in Figure 3B). This is reasonable as the correct classification between gray and white matter voxels is not critical when using T2*-images at low spatial resolution.
- In order to model high signal values of the CSF in the ventricles and around the cortex, an explicit CSF prior was included (CSF in Figure 3B).
- Because there is maximal uncertainty about the a priori spatial locations of CMBs, that is, they may appear anywhere in the brain [1], [19], a flat prior was used (CMB in Figure 3B). This was constructed by having non-null values (e.g. prior = 0.1) inside the brain and zeros outside the brain. It models all voxels that cannot be considered as “normal” in the mixture of Gaussians algorithm (similar to the “rejection class” principle suggested by previous work [20]). This is the empirical prior that is optimised iteratively in the next steps.
- To take into account low signal intensity voxels around the brain (i.e. the skull) that might be similar to the intensities of interest in CMBs, a fourth tissue probability map (“skull” in Figure 3B) was explicitly included.
- The soft tissue outside the cortex with relatively high intensities (e.g. scalp and eyes) was modelled with an additional tissue probability map (“scalp” in Figure 3B).
- All voxels outside the subject's head (air/background) were explicitly modelled with another prior (noted “other” in Figure 3B); this is included by default in the unified segmentation procedure of SPM8 [5].

The unified normalisation-segmentation model combines tissue class, intensity bias and nonlinear warping into the same probabilistic models that are assumed to generate subject-specific images (see equation (14) in [5]). Hence, it was critical to ensure that the mixture of Gaussians would model the appropriate intensities of the tissue class expected at the locations coded in the priors. Following an initial test of the algorithm (results not shown here), we implemented two empirical constrains on the mixture of Gaussians during the optimisation of its parameters using the Expectation-Maximisation algorithm. This was to minimise the impact of low contrast, partial volume in the thick slices and possible spatial distortions. We achieved this by forcing the mean of the Gaussians (equation (23) in [5]) for (i) CSF tissue to be higher than the mean of Gaussians of GWM (right-side to the dashed line of the histogram in Figure 3A), and (ii) CMBs to be less than the 50% of the mean of Gaussians of GWM (i.e. forcing the search for CMBs in the left-side to the dashed line of the histogram in Figure 3A).

All six priors were sampled to an isotropic 1.5 mm resolution. The unified model used the following number of Gaussians (3,2,2,3,2, and 4) to model each of the intensity distributions of GWM, CSF, CMBs, “skull”, “scalp” and the “other” tissues respectively. The rest of the normalisation-segmentation parameters were identical to our previous work (see for more details [6]).

#### 2. Optimisation of the empirical prior

The initial unified normalisation-segmentation on the index patient data resulted in different tissues classes which corresponded with the expected tissues, including the CMB class (Figure 4A). This image contained all the CMBs identified by an experienced rater for this patient. However, it also contained some artefacts (false positives) mimicking CMBs that needed to be removed. These artefacts were misclassified because their T2* intensities were comparable to CMBs (arrows in Figure 4B). These “mimics” include calcification or iron deposits in the basal ganglia, dentate nuclei, substantia nigra, and brainstem; flow voids of cerebral vessels and air/bone susceptibility artefacts particularly at the edges of the frontal or temporal lobes, or cerebellum.

To minimise false positives we used the following morphological operations [18]. First, the CMB class was converted to a binary image using a 0.2 probability threshold. Second, we set an upper limit for the size of a cerebral microbeed to differentiate them from “macrobleeds” (e.g. lobar haemorrhage). We set an upper diameter limit for a CMBs of 8 mm; a previous study suggests that intracerebral haemorrhage volume (at least in CAA) has a bimodal distribution with a cut off of about 5.7 mm diameter [7], [21], [22]. In order to take into account possible partial volume effects, we used a cut-off volume of 0.675 cm^3^ corresponding to a cylinder of maximum 2-slices high by 8 mm diameter. All regions with larger volumes (>0.675 cm^3^) assessed by morphological granulometry (see pages 318–327 of Ref. [18]) were excluded. It is also possible to introduce a lower limit for the size of CMBs in order to match the thresholds used in the manual procedure (see below). To do that, only CMBs that are at least two contiguous voxels in size were considered (i.e. single voxel CMBs (volume<0.006 cm^3^), if any, were excluded). Third, all regions of the “skull” class, including the artificial hypointensities around the cortex (e.g. air-bone susceptibility effects at the skull base), were used as a mask and removed from the CMB class. Fourth, because we were not interested in hypointenisties within CSF, we excluded all voxels and their nearest neighbours that showed a reasonably high probability (>0.5) of being in the CSF tissue class. Finally, we created an additional mask of irrelevant voxels by including all voxels that had been identified as *false positive* CMBs (e.g. from basal ganglia calcification [23] or air-bone susceptibility artefacts) in any of our 44 healthy controls. This was achieved by running the whole automated procedure on each healthy control separately using the same parameters and then grouping any identified voxels as CMBs in one map (that we referred to as the artefact mask). Note that air-bone susceptibility effects were the dominant source of artefacts (false positives) over our 44 controls, in particular at the edges of the inferior temporal, orbitofrontal and posterior fossa regions. Hypointense areas in patients classified as CMBs by our method were discarded if they overlapped with this artefact mask. These different morphological operations (i.e. binarisation, granulometry and masking) were *hard-coded* by default in our method and produced a refined image of the CMB class (see schematic illustration of all steps in Supplementary Material S1).

#### 3. Unified normalisation-segmentation: second iteration

**Figure 5:**
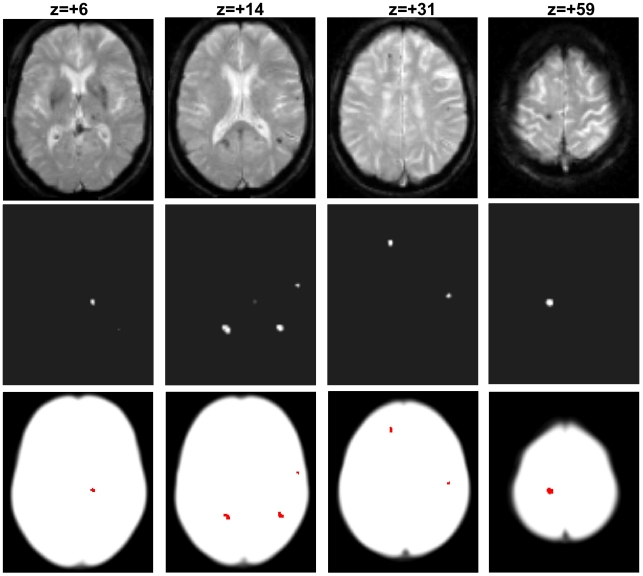
Illustration of genuine cerebral microbleeds. Top: axial T2*-weighted slices at different z-coordinates. Middle: final output images from the second iteration of MIDAS showing the microbleeds identified in white. Bottom: visualisation of the same cerebral microbleeds in red on a white background whole-brain mask.

The refined version of the CMB class was then used with the other unchanged classes (GWM, CSF, “skull”, and “scalp”) as new priors for unified normalisation-segmentation of the original T2*-weighted image. This second iteration was more specific for identifying true CMBs (Figure 5), as the influence of artificial hypointensities had been minimised. The identified hypointensities appeared as continuous probability values in the CMBs class varying from 0 to 1 (Figure 5 middle row); these values represent the likelihood that a voxel is part of a CMB rather than one of the other four tissue classes. It is possible to threshold this image to generate a binary CMB map (example with CMBs shown in red, lowest row Figure 5, using typically a threshold of 0.5), and calculate both their exact location in stereotaxic MNI space, and volume (not shown).

MIDAS takes less than 3 minutes to run per patient (PC 64-bit, 3.2 GHz Intel CPU, 12 GB RAM). An additional 5–10 minutes on average were needed for the semi-automated approach to manually exclude the obvious artefacts that were seen in some patients (see Results section above).

### Manual identification of cerebral microbleeds: reference standard

The GRE T2*-weighted MRI images were displayed in semi-dark conditions using an Agfa IMPAX PACS system, and assessed manually by a clinical neurologist (SMG) who was trained in CMB rating by an experienced consultant neuroradiologist (HRJ). The presence, number and anatomical distribution of CMBs was rated using the validated Microbleed Anatomical Rating Scale (MARS) [3]. The rater was blinded to clinical data and the results of automatic CMB detection procedure. Definite CMBs were defined as small, rounded or circular, well-defined hypointense lesions within brain parenchyma with clear margins raging from 2 mm to 10 mm in size. Care was taken to exclude all CMB mimics using all available imaging, including axial T2-weighted fast spin echo images that were acquired in the same session as the GRE T2* images. In particular, flow voids in blood vessels were excluded by their location in cerebral sulci, their visibility on the T2-weighted images, and their lack of “blooming” on T2*-weighted images. The inter-rater agreement for the presence of definite CMBs identified in any brain location using MARS was kappa = 0.68 [3].

The manual identification is considered here as the reference standard to define the true positives. All identified abnormalities from MIDAS were classified by a trained observer (MAK) as true positives or false positives (artefacts), without reference to the manual ratings. Cases of uncertainty (in 5 patients) were decided by consensus (MAK and DJW). Agreement between the manual and the automated method was assessed by the intraclass correlation coefficient (ICC) and Kappa statistics [24], either over the whole brain or in separate brain regions (e.g. lobar versus deep/infratentorial). The interpretation of Kappa values was based on the following definitions: <0.2, 0.2–0.4, 0.4–0.6, 0.6–0.8, >0.8 for poor, fair, moderate, good and excellent respectively (see Table II of ref. [25]). All *post hoc* analyses were performed with the Statistical Package for the Social Sciences (SPSS, v. 16.0, IBM).

## Supporting Information

Supplementary Material S1 (PDF)
