# Supplementary material for: Microbleed Detection Using Automated Segmentation (MIDAS): A New Method Applicable to Standard Clinical MR Images
Source: PLoS One. 2011 Mar 23;6(3):e17547. doi: 10.1371/journal.pone.0017547 (PMC3063172; doi:10.1371/journal.pone.0017547)

Table S1: Summary of individual results for lobar microbleeds

MANUAL METHOD				AUTOMATED METHOD (MIDAS)				
**Case No.**	**MB Y/N**	**MB count**	**MB>1**	**MB Y/N**	**MB count**	**MB>1**	**Location / details of MB missed**	**Location / details of artefacts**
1	Y	1	N	Y	1	N	-	edge of temporal lobe
2	Y	1	N	N	0	N	small, partial volume (frontal)	-
3	N	0	N	N	0	N	-	-
4	Y	4	Y	Y	2	Y	small, partial volume (parietal)	edge of frontal lobe
5	N	0	N	N	0	N	-	-
6	Y	3	Y	Y	1	Y	small, partial volume (temporal)	temporal lobe vessel
7	N	0	N	N	0	N	-	-
8	Y	1	N	N	0	N	small, partial volume (frontal)	-
9	Y	2	Y	Y	1	Y	small, partial volume (parietal)	-
10	N	0	N	N	0	N	-	-
11	Y	3	Y	Y	2	Y	partial volume (parietal), small (temporal)	edges of frontal and occipital lobes
12	N	0	N	N	0	N	-	edge of temporal lobe
13	N	0	N	N	0	N	-	haemorrhage within an infarct
14	Y	1	N	N	0	N	partial volume (occipital)	-
15	N	0	N	N	0	N	-	edge of frontal lobe
16	Y	11	Y	Y	7	Y	partial volume (parietal)	-
17	Y	1	N	N	0	N	small, partial volume (parietal)	-
18	Y	1	N	Y	1	N	-	edge of frontal lobe
19	Y	14	Y	Y	14	Y	-	edge of temporal lobe
20	Y	1	N	Y	1	N	-	-
21	Y	1	N	Y	1	N	-	-
22	Y	1	N	Y	1	N	-	-
23	Y	2	Y	Y	1	Y	masked (temporal)	edge of frontal lobe
24	Y	7	Y	Y	2	Y	small (temporal, parietal)	-
25	Y	1	N	Y	1	N	-	edges of frontal and temporal lobes
26	Y	1	N	Y	1	N	-	-
27	Y	14	Y	Y	8	Y	small (occipital, parietal)	edges of frontal and parietal lobes
28	Y	1	N	N	0	N	masked (temporal)	-
29	N	0	N	N	0	N	-	edges of temporal and occipital lobes
30	Y	1	N	Y	1	N	-	edge of frontal lobe

MB = microbleed; Y/N= Yes/no for presence of microbleeds

Legend: comparison between the manual reference standard and MIDAS for identification of lobar microbleeds in each patient.

Table S2: Summary of individual results for deep and infratentorial microbleeds

MANUAL METHOD				AUTOMATED METHOD (MIDAS)			
Case No.	MB Y/N	MB count	MB>1	MB Y/N	MB count	Location / details of MB missed	Location / details of artefacts
1	N	0	N	N	0	-	cerebellum, brain stem
2	Y	1	N	N	0	small (thalamus)	basal ganglia
3	Y	1	N	N	0	masked (basal ganglia)	-
4	N	0	N	N	0	-	basal ganglia
5	Y	1	N	N	0	small (thalamus)	-
6	N	0	N	N	0	-	-
7	Y	1	N	N	0	masked (basal ganglia)	periventricular white matter
8	N	0	N	N	0	-	-
9	N	0	N	N	0	-	cerebellum, basal ganglia
10	Y	1	N	N	0	small (basal ganglia)	-
11	N	0	N	N	0	-	cerebellum
12	Y	1	N	Y	1	-	basal ganglia, substantia nigra
13	Y	1	N	N	0	masked (basal ganglia)	edges of cerebellum
14	N	0	N	N	0	-	basal ganglia, brainstem
15	Y	1	N	N	0	masked (basal ganglia)	cerebellum, basal ganglia
16	Y	2	Y	Y	1	partial volume (thalamus)	-
17	Y	5	Y	N	0	masked (thalamus, internal capsule)	cerebellum
18	N	0		N	0	-	basal ganglia, cerebellum
19	Y	3	Y	Y	1	small (cerebellum), big (corpus callosum)	cerebellum
20	N	0	N	N	0	-	-
21	N	0	N	N	0	-	-
22	Y	7	Y	Y	5	masked (thalamus)	-
23	Y	2	Y	Y	0	masked (brainstem, thalamus)	basal ganglia, cerebellum
24	Y	1	N	N	0	masked (dentate nucleus)	-
25	Y	4	Y	Y	2	masked (lentiform), small (thalamus)	brain stem
26	N	0	N	N	0	-	-
27	Y	7	Y	Y	1	small (brainstem), masked (basal ganglia, thalamus)	-
28	N	0		N	0	-	periventricular
29	Y	2	Y	Y	0	masked (thalamus, crebellum)	-
30	N	0		N	0	-	basal ganglia

MB = microbleed; Y/N= Yes/no for presence of microbleeds

Legend: comparison between the manual reference standard and MIDAS for identification of deep and infratentorial microbleeds in each patient.

Figure S1: illustration of the different morphological procedures during the optimization of the empirical CMB prior. See Methods section for more details.

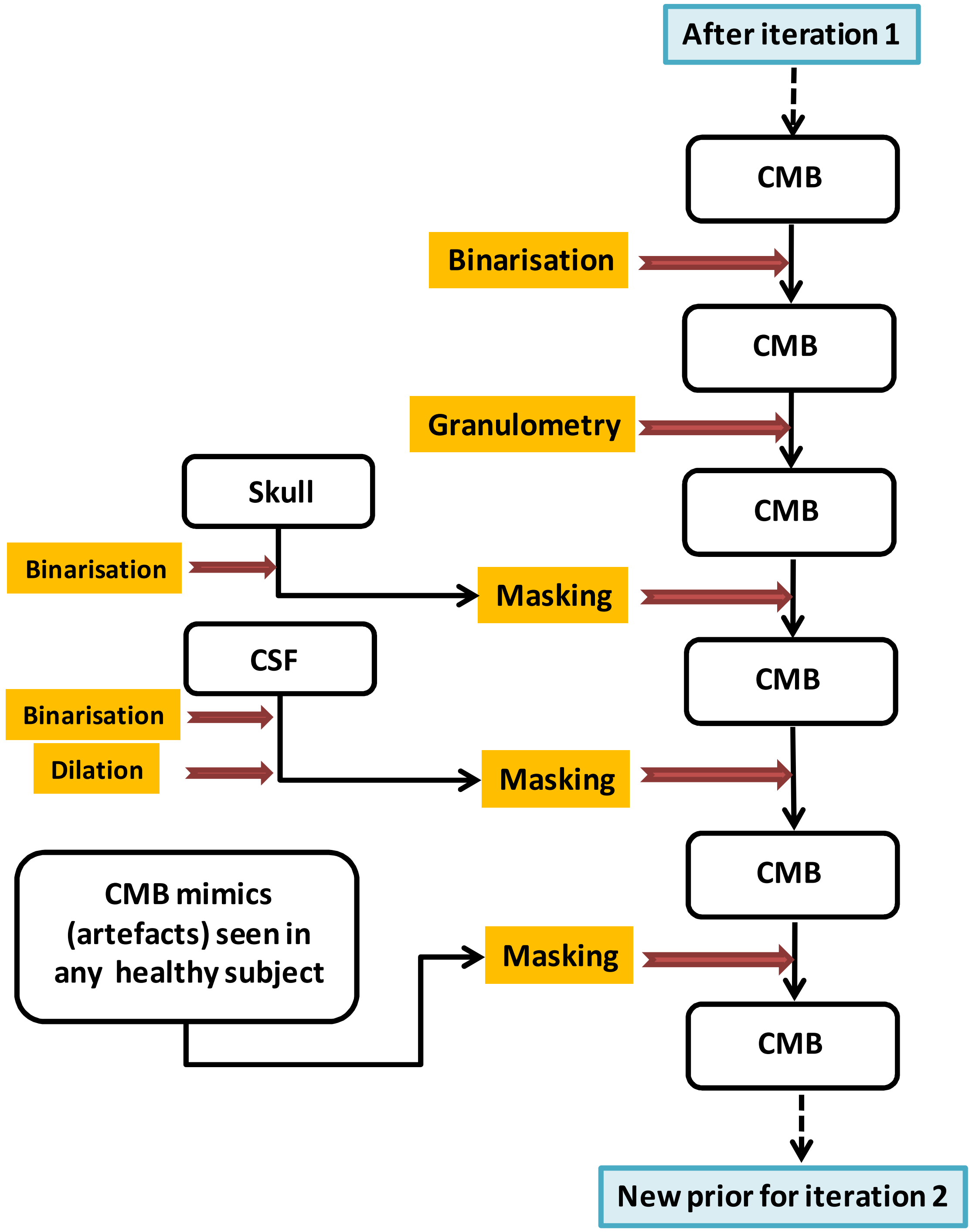

Supplement: Supplementary Material S1 — (PDF) [file pone.0017547.s001.pdf]
